# Interferon Regulatory Factors in Alcohol-Associated Liver Disease: Cell-Type Programs, Danger Signaling, and Therapeutic Opportunities

**DOI:** 10.3390/cimb48010092

**Published:** 2026-01-16

**Authors:** Haibo Dong, Wei Guo, Zhanxiang Zhou

**Affiliations:** 1 Center for Translational Biomedical Research, University of North Carolina at Greensboro, North Carolina Research Campus, Kannapolis, NC 28081, USA; h_dong@uncg.edu (H.D.); w_guo2@uncg.edu (W.G.); 2 Department of Nutrition, University of North Carolina at Greensboro, Greensboro, NC 28081, USA

**Keywords:** interferon regulatory factors (IRFs), oxidative stress, LPS, cGAS–STING signaling, toll-like receptors signaling, mitochondrial dysfunction, hepatic inflammation, PANoptosis, post-translational regulation, gut-liver axis

## Abstract

Alcohol-associated liver disease (ALD) contributes substantially to the global burden of cirrhosis and liver-related mortality, driven by ethanol metabolism, oxidative stress, and dysregulated immune signaling. Despite rapidly growing evidence implicating interferon regulatory factors (IRFs) in ALD pathogenesis, an integrated framework linking ethanol-induced danger signals to cell-type-specific IRF programs is lacking. In this comprehensive review, we summarize current knowledge on IRF-centered signaling networks in ALD, spanning DAMP–PAMP sensing, post-translational IRF regulation, and downstream inflammatory, metabolic, and fibrogenic outcomes across various cell types in the liver, including hepatocytes and immune-related cells such as Kupffer cells, monocyte-derived macrophages, dendritic cells, T cells, hepatic stellate cells (HSC), and neutrophils. We also focus on how ethanol-driven DAMP and PAMP signals activate TLR4, TLR9, and cGAS–STING pathways to engage a coordinated network of IRFs—including IRF1, IRF3, IRF4, IRF5, IRF7, and IRF9—that collectively shape inflammatory, metabolic, and cell-fate programs across hepatic cell populations. We further highlight emerging therapeutic strategies such as STING/TBK1 inhibition, NETosis blockade, IL-22-based epithelial repair, and JAK-STAT modulation that converge on IRF pathways. In summary, this review outlines how IRFs contribute to ALD pathogenesis and discusses the potential implications for the development of targeted therapies.

## 1. Introduction

Alcohol-associated liver disease (ALD) encompasses a clinical and pathological spectrum ranging from steatosis to steatohepatitis, fibrosis, and cirrhosis, representing a major contributor to global liver-related morbidity and mortality [1]. Despite substantial progress in delineating its pathobiology, therapeutic advances for ALD remain constrained by its inherently multifactorial nature, in which metabolic perturbations, dysregulated immune signaling, and gut–liver axis dysfunction converge to drive disease progression. Ethanol metabolism through alcohol dehydrogenase (ADH) and cytochrome P450 2E1 (CYP2E1) pathways generates the toxic metabolite acetaldehyde, as well as reactive oxygen and nitrogen species (ROS, RNS), leading to mitochondrial dysfunction, lipid peroxidation, and protein or DNA adduct formation [2,3,4]. These oxidative events convert hepatocytes from passive targets into active sources in inflammation by releasing damage-associated molecular patterns (DAMPs) and pro-inflammatory mediators that activate Kupffer cells and recruit neutrophils and monocyte-derived macrophages to the liver [5,6,7]. Over time, these reciprocal inflammatory and metabolic disturbances gradually reinforce each other and drive fibrogenesis [8]. Therefore, understanding how early metabolic and oxidative stress evolves into chronic immune activation remains a central challenge in the pathogenesis of ALD.

Interferon regulatory factors (IRFs 1–9) were initially identified as transcriptional mediators of antiviral interferon responses in mammals [9]. However, accumulating evidence suggests that they also integrate immune, metabolic, and cell death pathways in various pathological conditions. Within the hepatic microenvironment, these IRFs coordinate distinct yet interconnected programs. For instance, IRF1, IRF5, and IRF8 promote classical macrophage activation and pro-inflammatory cytokine production [10,11,12,13,14,15]. IRF3 acts as a key sensor–effector linking mitochondrial and cytosolic nucleic-acid stress to type I interferon induction, yet under chronic stimulation, it can switch to a pro-apoptotic role [16,17,18,19,20]. IRF7, the master regulator of type I interferon (IFN-I) production, is activated through both MyD88-dependent (e.g., endosomal TLR7 and TLR9 in plasmacytoid dendritic cells) and MyD88-independent (e.g., TLR3–TRIF or TLR4–TRIF in endosomes) pathways [21,22]. Meanwhile, IRF4 and IRF8 govern dendritic-cell differentiation and T-helper cell polarization [23,24], whereas IRF6 and IRF9 are emerging as regulators of hepatic lipid metabolism. In non-alcoholic metabolic models, IRF6 restrains PPARγ-linked lipogenic programs, while IRF9 can interact with PPARα to support fatty-acid oxidation gene expression [25,26]. Taken together, current data suggest that IRFs link pattern-recognition receptor (PRR) signaling to metabolic stress and inflammatory progression in the liver. In ALD, chronic ethanol exposure disrupts this transcriptional balance, amplifying pro-inflammatory and fibrogenic programs while weakening cytoprotective and reparative mechanisms.

Recent studies have further clarified the upstream sensors that activate IRFs in alcohol-induced liver injury. The TLR4 signaling pathway connects gut-derived endotoxin exposure to IRF3 and IRF7 activation in hepatocytes and Kupffer cells [27,28]. Sustained or excessive IRF3 signaling activation shifts its activity toward non-transcriptional, pro-apoptotic functions and promotes necro-inflammatory injury [17,20]. Mitochondrial stress and cytosolic DNA leakage engage the cGAS–STING–IRF3 axis, amplifying hepatocyte death and inflammatory gene expression through intercellular propagation [29]. Beyond its canonical transcriptional role, IRF3 also executes a non-transcriptional apoptotic program (RIPA) via engagement with mitochondrial Bax, in which stress cues promote IRF3 translocation to mitochondria and its co-localization/interaction with Bax, thereby triggering downstream mitochondrial apoptotic signaling with caspase-9 and caspase-3 activation and PARP cleavage that enhances hepatocellular death and skews Ly6C^low monocytes toward a pro-inflammatory phenotype under sustained ethanol stress [20]. In parallel, oxidative and neuropeptide signaling activate IRF1 in macrophages, linking NOX2-derived ROS to inflammasome activation and cytokine release, while autophagic degradation of IRF1 serves as a counter-regulatory mechanism [10,30]. Moreover, TLR7–IRF7 signaling contributes to interferon amplification and may participate in gut–brain–liver communication under chronic alcohol exposure [31]. Collectively, these findings underscore that ALD is not solely a metabolic or toxicity-mediated injury, but an immune–metabolic disease in which IRF-centered transcriptional and non-transcriptional pathways play key integrative roles.

Current evidence indicates that ethanol metabolism, gut-derived microbial cues, and mitochondrial danger signaling converge on a restricted set of PRR pathways—most prominently TLR4, TLR7/9, and cGAS–STING—to activate IRF-dependent transcription in hepatocytes and hepatic immune cells. These coordinated stress and innate immune responses help clarify why ALD evolves from simple steatosis to inflammatory and fibrotic injury. However, an integrated framework that connects DAMP–PAMP signatures to distinct IRF nodes and how these circuits connect to cell-specific injury mechanisms and therapeutic opportunities remains to be elucidated. To address this gap, this review specifically: (i) maps ALD-relevant DAMP–PAMP–PRR pathways to discrete IRF signaling modules; (ii) dissects cell-type-specific IRF programs across hepatocytes and hepatic immune cells; and (iii) links these mechanistic insights to emerging therapeutic strategies for ALD that act through direct or indirect modulation of IRF activity.

## 2. Alcohol-Driven DAMPs and PAMPs in IRF Ignition

Ethanol metabolism in the liver is primarily mediated by ADH, CYP2E1, and catalase, while sustained activation of these enzymes profoundly disturb hepatocellular redox homeostasis [2,32]. Their combined activity reduces the NAD^+^/NADH ratio, suppresses fatty-acid oxidation, and drives mitochondrial ROS/RNS overproduction [33]. CYP2E1-dependent lipid peroxidation further generates reactive aldehydes such as 4-hydroxynonenal (4-HNE) and malondialdehyde (MDA), which form stable adducts with proteins, lipids, and DNA [34,35]. Although 4-HNE and MDA are not known to directly bind or modify IRF proteins, they exert strong upstream effects by promoting mitochondrial depolarization, ER stress, and hepatocellular necrosis—events that trigger the release of mitochondria-derived DAMPs, including mtDNA, mt-dsRNA, TFAM-bound nucleoids, HMGB1, and DAMP-rich extracellular vesicles [36,37,38]. These endogenous DAMPs constitute the primary sterile signals that feed into IRF-activating pathways such as TLRs, and cGAS–STING, forming one of the major upstream driving forces in ALD.

At the same time, chronic alcohol consumption disrupts intestinal epithelial tight junctions and reshapes the gut microbiota, leading to increased gut permeability and translocation of multiple pathogen-associated molecular patterns (PAMPs) into portal blood [39,40]. These PAMPs include lipopolysaccharide (LPS), bacterial DNA, flagellin, and fungal β-glucans, which can activate hepatic TLR4, TLR9, TLR3, and dectin-1 pathways [27,28,41,42,43]. PAMP-initiated signaling through MyD88 or TRIF provides a sustained microbial source of IRF-activating stimuli in ALD. Together, the mitochondrial DAMPs released from damaged hepatocytes and the microbial PAMPs originating from a permeable gut provide two major sources of danger signals that converge on IRF-dependent transcriptional and non-transcriptional programs, initiating ALD-related immune activation.

### 2.1. DAMP-Driven Activation of IRF Pathways

Alcohol-induced oxidative and ER stress destabilize mitochondrial structure and initiate the release of mitochondrial DNA (mtDNA) and TFAM-bound nucleoids into the cytosol or circulation [36,44,45,46,47]. These nucleic acid DAMPs constitute a major mechanistic bridge between ethanol-induced mitochondrial injury and IRF activation. Cytosolic mtDNA engages the cGAS–STING pathway [48,49,50], whereas endosomal mtDNA activates TLR9 [46]. Both sensing routes converge on TBK1/IKKε, leading to phosphorylation of IRF3 and IRF7 and subsequent induction of type-I interferons and interferon-stimulated genes (ISGs) [51,52]. In hepatocytes, cGAS–STING–IRF3 signaling integrates mitochondrial and ER stress into apoptotic circuits, and gap-junction transfer of cGAMP further propagates IRF3 activation to adjacent hepatocytes, creating a multicellular amplification wave [17,29].

Additional DAMP species, such as HMGB1, heat-shock proteins [53], and extracellular vesicles with lipids, proteins, chemokines, and nucleic acids [54,55,56,57,58,59,60,61,62,63], are sensed by pattern recognition receptors (PRRs) on the surface or within the cells [64,65]. Although these agonists are classically associated with NF-κB and inflammasome activation, TRIF–TBK1 signaling, the downstream targets of TLRs, can also activate IRF3 and IRF7 [66,67,68,69,70], particularly in the presence of concurrent mitochondrial stress. DAMPs-induced cytokines, including TNF, IL-1 family members, and CCL2, further remodel transcriptional states of macrophages, dendritic cells, or other immune cells toward IRF1- and IRF5-dominant programs linked to nitric-oxide production, chemotaxis, and stellate-cell priming [71,72]. While lipid peroxidation products such as 4-HNE and MDA are abundant in ALD, current evidence suggests that their influence on IRFs is indirect—acting through upstream stress pathways rather than via direct IRF modification.

### 2.2. PAMP-Driven Activation of IRF Pathways

Complementing sterile danger signals, chronic alcohol consumption increases exposure of liver to gut-derived PAMPs, which form a second major input into IRF pathways. Among these, LPS is the best-characterized mediator, activating TLR4 in Kupffer cells, liver sinusoidal endothelial cells, and stellate cells [40]. The MyD88 arm of TLR4 primarily drives NF-κB–dependent inflammatory cytokines, whereas the TRIF branch recruits TBK1/IKKε and phosphorylates IRF3 and IRF7, providing a microbial entry point into IFN-I and ISG pathways [17,27,29].

Other microbial molecules—including bacterial DNA (TLR9) [73], lipoteichoic acid (TLR2) [39], flagellin (TLR5) [74,75] and fungal β-glucans (CLEC7A/Dectin-1) [76]—further reinforce IRF activation across hepatic parenchymal and myeloid compartments. In macrophages, cytokines and oxidative cues downstream of PAMP sensing activate IRF1 and IRF5, linking NOX2-derived ROS production [10], inflammasome assembly [77], and cytokines/chemokine induction [78] to the “stress-responsive” IRF module [79]. Thus, alcohol-induced gut leakage provides a continuous microbial input that maintains IRF1/IRF5 activation via TLR2/5/9 signaling and reinforces IRF3/IRF7 signaling through TLR4–TRIF and related pathways.

### 2.3. IRF-Linked Inflammatory Cell Death and Feed-Forward Amplification

Beyond transcriptional responses, recent work highlights a role for IRF signaling in amplifying tissue injury through inflammatory cell death. IRF1 and IRF9 transcriptionally upregulate Z-DNA binding protein 1 (ZBP1), a cytosolic nucleic acid sensor that drives PANoptosis—a programmed blend of pyroptosis (P), apoptosis (A), and necroptosis (N). Ethanol exposure induces IRF1/IRF9-dependent ZBP1 expression in macrophages, Kupffer cells, monocytes, and hepatocytes, and activation of this pathway results in membrane rupture and release of DAMPs such as HMGB1 [80]. These DAMPs, in turn, intensify IRF3/IRF7 signaling, creating a powerful feed-forward loop in which IRFs not only sense innate immune cues but also promote further DAMP release through cell-death mechanisms.

**Figure 1:**
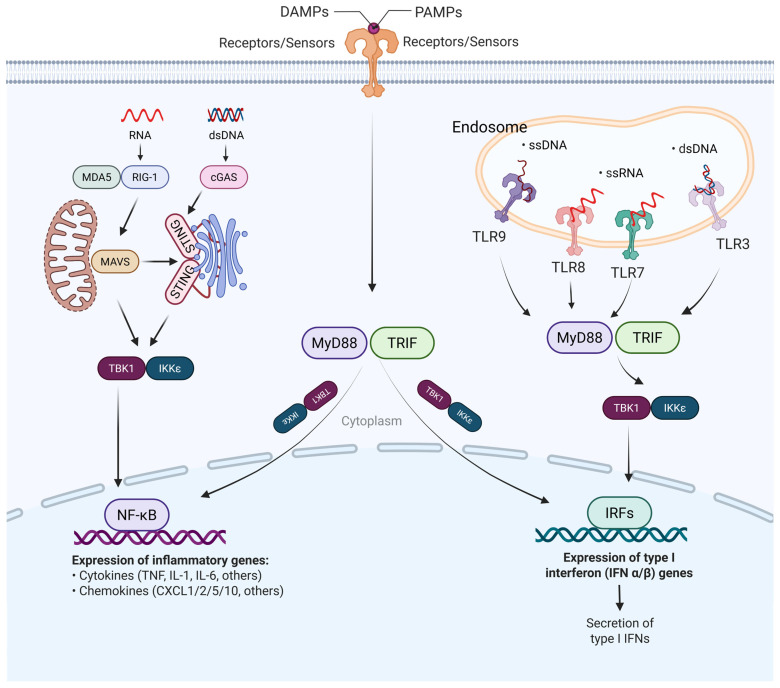
Ethanol-induced DAMP and PAMP signaling converge on NF-κB and IRF activation in alcohol-associated liver disease (ALD). Ethanol metabolism in the liver promotes hepatocellular injury and barrier dysfunction, leading to the release of damage-associated molecular patterns (DAMPs), including extracellular DNA, RNA, and HMGB1, from injured hepatocytes, and facilitates the translocation of pathogen-associated molecular patterns (PAMPs), such as lipopolysaccharide (LPS) and microbial nucleic acids, from the gut into the portal circulation. These danger signals are sensed by hepatic pattern-recognition receptors (PRRs) in distinct subcellular compartments. Cytosolic nucleic acids activate RIG-I and MDA5 or the cGAS–STING axis, whereas endosomal ssRNA, ssDNA, and dsRNA engage TLR7/8, TLR9, and TLR3, respectively. Downstream adaptor signaling bifurcates through MyD88 and TRIF, leading to activation of TBK1 and IKKε. MyD88-dependent pathways preferentially activate NF-κB to induce inflammatory cytokines and chemokines, including TNF, IL-1β, IL-6, and CXCL family members, while TRIF-dependent signaling promotes IRF activation and type I interferon gene transcription. The convergence of NF-κB- and IRF-mediated programs establishes a self-amplifying innate immune network that drives hepatic inflammation and tissue injury in ALD.

Collectively, these findings indicate that ALD engages a defined set of danger signals that converge on TLRs, NOD-like receptors, and cGAS–STING to initiate IRF-driven transcription. Importantly, these PRR pathways map onto distinct IRF nodes: first, mtDNA, oxidized nucleic acids, and dsRNA preferentially activate IRF3 and IRF7 via cGAS–STING and TLR3/9; second, LPS/TLR4 signaling engages IRF1 and IRF5 through MyD88–IRAK1–TRAF6; and third, bacterial peptidoglycan sensed by NOD2 induces IRF4-dependent tolerogenic programs in liver pDCs. Figure 1 summarizes how ethanol-induced DAMPs and gut-derived PAMPs activate TLR4–TRIF–TBK1 and cGAS–STING cascades to converge on NF-κB and IRF signaling in ALD. To extend this overview, Table 1 lists the major DAMP/PAMP cues and their corresponding PRR–IRF modules.

## 3. IRF Signaling Architecture and Control by Post-Translational Modifications

Interferon regulatory factors (IRFs 1–9) share a conserved two-domain organization that enables them to convert innate immune signals into tailored transcriptional outcomes. Their N-terminal DNA-binding domain (DBD) recognizes interferon-stimulated response elements (ISREs), whereas the C-terminal IRF-association domain (IAD) governs homo- and heterodimerization with IRFs, STATs, Spi-1, CREB, BATFs, and co-activators such as CBP and p300 [120]. This modular design allows IRFs to combine with various upstream inputs to generate specific transcriptional responses: upstream pattern-recognition receptors (PRRs) determine which IRFs become activated, while dimer composition and co-factor recruitment dictate promoter selectivity and gene-expression amplitude. The accuracy of these transcriptional programs relies on post-translational modifications (PTMs), which collectively determine each IRF’s stability, subcellular localization, chromatin engagement, and transcriptional activity [121].

A network of kinases, E3 ligases, SUMO enzymes, and acetylation/deacetylation machinery coordinates IRF activation in response to cellular stress. TBK1 and IKKε are the canonical IRF3/IRF7 kinases that phosphorylate C-terminal serine clusters, enabling dimerization, nuclear entry, and induction of type I interferons [66,122]. Stress-activated MAPKs such as p38 enhance the transcriptional output of IRF1/3 [123,124], reinforcing inflammatory programs. Ubiquitin linkage specificity further refines IRF signaling: K63-linked ubiquitination enhances activation of IRF1 [125,126], IRF5 [127,128], and IRF7 [129,130], whereas K48-linked ubiquitination mediates proteasomal degradation of IRF1 [125,131], IRF3 [132,133], IRF7 [134], and IRF8 [135], thereby enforcing resolution once danger signals subside. SUMOylation generally imposes negative regulation, repressing IRF1 [136], IRF2 [137], IRF3 and IRF7 [138,139] activity, while acetylation by p300/PCAF increases chromatin accessibility and stabilizes IRF–DNA interactions [140]. Counterbalancing this, NAD^+^-dependent deacetylases such as SIRT1 restrain IRF acetylation and dampen excessive inflammatory output [141]. Collectively, these PTM systems determine whether IRFs promote antiviral defense, inflammatory activation, or programmed cell death.

Within the context of ALD, chronic oxidative stress, altered NAD^+^ metabolism, and persistent stimulation reconfigure this PTM landscape in ways that gradually direct IRF signaling toward pathogenic outputs. Ethanol metabolism decreases the NAD^+^/NADH ratio and reduces SIRT1 activity [142,143,144] and thus may favor p300/PCAF-mediated acetylation of IRF3 and IRF7 [145], which prolongs ISG expression beyond the adaptive window. At the same time, K48-linked ubiquitination serves as a major resolution mechanism for IRF signaling by promoting the proteasomal degradation of IRF1 [125,131]. A recent finding further implicates FBXW7, an E3 ubiquitin ligase, in this regulatory axis: silencing FBXW7 reverses alcohol-induced decrease in IRF1, whose degradation contributes to suppression of NMNAT1, a key nuclear NAD^+^ biosynthetic enzyme [146]. This signaling mechanism creates a feed-forward loop in which ethanol impairs both upstream NAD^+^ production and downstream IRF resolution, thereby reinforcing a pro-inflammatory, low-repair transcriptional state.

In addition, activation of TBK1/IKKε and TLR4–TRIF signaling leads to sustained phosphorylation of IRF3 and IRF7, supporting ISG responses in hepatocyte–macrophage circuits [27,29]. However, under chronic ER–mitochondrial stress, the same phosphorylation axis shifts IRF3 toward its non-transcriptional pro-apoptotic function through Bax interaction, driving hepatocyte death, particularly during the early stages of ALD [17,20]. Gap-junction propagation of cGAMP further allows IRF3 activation to spread radially across adjacent hepatic cells [29], amplifying inflammatory signals in ALD.

In immune cells, alcohol impairs autophagy and mitophagy, limiting the degradation of IRF1 and damaged mitochondria [30]. As a result, the IRF1 protein becomes more stable and facilitates nuclear translocation, augmenting CCL5 and CXCL10 expression, two important chemokines involved in liver inflammation. This pathway is associated with a Lys63 (K63)-linked polyubiquitination pathway, which has been reported to be crucial for ALD development [147]. In parallel, alcohol also enhances the transcriptional pool of IRF1 through a p300-mediated H3K27ac mechanism at the GRP/GRPR promoters [10]. Together, these alterations create a dual reinforcement—reduced degradation and increased transcription—that amplifies IRF1-driven inflammatory output. Similar to IRF1, IRF3, and IRF-7, IRF-5 also requires phosphorylation-induced nuclear translocation to activate type I IFNs. Although direct mapping of IRF5 PTMs in ALD remains incomplete, several key regulatory mechanisms are well established in macrophages. IRF5 activation depends on phosphorylation of its C-terminal serine cluster, which is mediated by TRAF6 and the SLC15A4–TASL–MyD88 adaptor complex downstream of endosomal TLR7/8/9 signaling [148] or is activated by K63-linked polyubiquitination in TLR7/9-MyD88 signaling [127]. Taken together, the convergence of altered phosphorylation dynamics, defective ubiquitin-mediated degradation, and dysregulated acetylation creates an IRF signaling state characterized by prolonged activation, impaired resolution, and enhanced cytotoxicity—an architecture central to the inflammatory progression of ALD.

## 4. IRF Signaling Across Hepatic Cell Populations in Alcohol-Associated Liver Disease

ALD engages IRF pathways in a highly cell-type-specific manner, reflecting distinct sensing functions and transcriptional wiring of hepatocytes, Kupffer cells, monocyte-derived macrophages, dendritic cells, T cells, neutrophils, and hepatic stellate cells. Ethanol metabolism generates DAMPs and promotes gut-derived PAMP translocation, activating PRRs such as TLR4, TLR7/9, RIG-I, and cGAS–STING. These upstream cues converge on IRF1-IRF9, driving divergent outcomes—from hepatocellular death and sterile inflammation to macrophage polarization, DC–T cell imbalance, and HSC activation. The following sections delineate the IRF circuitry unique to each hepatic cell population, and how these programs collectively orchestrate ALD progression.

### 4.1. Hepatocytes

Hepatocytes express a focused but functionally diverse set of IRFs, including IRF1, IRF2, IRF3, IRF5, IRF6, IRF7, IRF8, and IRF9, whereas IRF4 remains restricted to immune cells with no evidence supporting hepatocyte expression. IRF1 is constitutively active and provides intrinsic antiviral protection through baseline ISG induction [149], while IRF2 contributes to hepatocyte survival and stress regulation, including β-catenin signaling and ischemia–reperfusion tolerance [150,151]. IRF3 attenuates steatosis and insulin resistance by constraining IKKβ/NF-κB activity [152], and IRF7 is induced downstream of IRF3-dependent IFN-λ/IFN-β programs during viral or sterile stress [153]. IRF9, broadly expressed at baseline, is further elevated during metabolic or oxidative injury and promotes apoptosis via SIRT1 suppression and p53 acetylation [150]. Additional evidence demonstrates that IRF5, IRF6, and IRF8 are also play key roles in hepatocytes: IRF5 mediates Fas-induced apoptosis [154], IRF6 suppresses PPARγ to reduce lipid accumulation [25], and IRF8 aggravates steatosis through a BMAL1/PPARγ axis [155]. In ALD, hepatocyte IRF1, IRF3, IRF7, and IRF9 have been reported to be activated via cGAS–STING, TLR3/9, and JAK–STAT pathways, and ethanol-induced induction of IRF1 and IRF9 licenses ZBP1-dependent PANoptosis, releasing mtDNA, RNA, and HMGB1 that amplify IRF3/IRF7 signaling and propagate local inflammation [80]. Collectively, current evidence indicates that hepatocytes rely on an unexpectedly broad IRF repertoire to coordinate antiviral defense, metabolic control, and stress responses and that alcohol drives this system toward a highly inflammatory and DAMP-amplifying state during ALD progression.

### 4.2. Kupffer Cells and Monocyte-Derived Macrophages

Kupffer cells (KC) and monocyte-derived macrophages (MoMFs) express a broad IRF repertoire—IRF1, IRF2, IRF3, IRF4, IRF5, IRF7, IRF8, and IRF9—while direct evidence for IRF6 expression in hepatic macrophages is currently lacking. IRF1 and IRF2 form foundational regulators of macrophage activation thresholds: IRF1 amplifies IFN-γ/STAT1-dependent chemokines and adhesion molecules [156] and contributes to inflammatory KC responses in viral and sterile injury [157]. In contrast, IRF2 exerts a dual regulatory function: it restrains excessive type I IFN signaling to permit normal Ly6C^−^MHCII^+^ MoMF differentiation, and simultaneously potentiates IFN-γ responsiveness in resident KCs to maintain steady-state MHCII expression, thereby supporting antigen presentation and KC identity [158]. IRF2 also restrains TLR–IFN sensitivity and controls macrophage apoptosis via STAT1/3–caspase-1 pathways [159,160]. During alcohol exposure, IRF1 and IRF9 are strongly induced and transcriptionally activate ZBP1, licensing KC/MoMF PANoptosis and release of mtDNA, RNA, and HMGB1 that enhance IRF3/IRF7 signaling [80,161]. IRF1 also mediates ferroptotic KC death under inflammatory stress [162]. IRF3 and IRF7 constitute the core interferon module: TLR4–TRIF–TBK1 activation drives IRF3-dependent TNF and IFN-β production in ALD [27,163,164,165], whereas IRF7 is expressed in human KCs and engages antiviral and inflammasome pathways downstream of TLR3/7/9 [166]. IRF5, activated through MyD88–IRAK1–TRAF6 K63-linked ubiquitination, promotes pro-inflammatory macrophage polarization [127]. IRF8 is required for yolk-sac-derived KC maturation [167] and exacerbates macrophage-dependent injury in liver ischemia–reperfusion [168]. IRF4, while absent in steady-state KCs, marks newly recruited BM-derived macrophages during inflammation and metabolic stress [169,170]. Together, these findings indicate that alcohol exposure shifts the macrophage IRF network toward a dominant IRF1/3/4/5/7/8/9-driven inflammatory state, thereby positioning KCs and MoMFs as central amplifiers of innate immune injury in ALD.

### 4.3. Dendritic Cells

Hepatic dendritic cells (DCs), including plasmacytoid DCs (pDCs) and conventional DC subsets (cDC1, cDC2)—express a broad interferon regulatory factor (IRF) repertoire in which IRF1, IRF4, IRF5, IRF6, IRF7, and IRF8 are prominent, whereas IRF2 and IRF9 exhibit more context-restricted activity. IRF4 and IRF8 cooperate in pre-cDCs to program terminal subset specification: cDC1 adopt a high-IRF8/low-IRF4 state that supports cross-presentation and type I IFN amplification, while cDC2 display high-IRF4/low-IRF8 and preferentially drive helper T-cell polarization and inflammatory cytokine production, as shown by lineage-tracing and transcriptional reprogramming studies where IRF8 deficiency converts cDC1 into cDC2-like cells [171,172,173,174,175,176]. Single-cell epigenomic profiling after viral vaccination further shows that cDC1 clusters are enriched for IRF8/IRF3/STAT motifs, whereas cDC2 and inflammatory DC clusters are dominated by AP-1–IRF composite elements and IRF4-linked programs, underscoring IRF-dependent chromatin wiring of DC antiviral and adjuvant responses [177,178,179]. Within pDCs, a refined IRF hierarchy governs antiviral and inflammatory bifurcation. IRF7 remains the master regulator of TLR7/9-driven type I IFN [180,181], but IRF3 and IRF5 execute distinct, non-redundant programs [182]. High-resolution transcriptomics from human pDCs revealed that IRF5 is selectively activated downstream of endosomal TLR7/8/9, whereas IRF3 is activated exclusively through RIG-I–like receptor (RLR) signaling—a strict pathway partitioning that produces divergent cytokine landscapes. TLR7/IRF5 activation drives pro-inflammatory mediators (IL-6, IL-12p70, TNF-α) and antigen-presenting programs, whereas RLR/IRF3 induces IFN-α subtypes and antiviral genes. These bifurcated responses explain how pDCs balance inflammatory adjuvanticity with classical type I IFN production. IRF5 also amplifies Th17-skewing chemokines (CCL3/4, IL-23), while IRF3 preferentially induces IFN-λ and antiviral interferon-stimulated gene programs, reinforcing antiviral positioning.

Liver-specific work highlights how these circuits shape tissue injury: liver pDC-derived IFN-α aggravates ischemia–reperfusion and transplant injury by inducing hepatocyte IRF1 and IL-15/IL-15Rα expression [183,184], whereas type I IFN downstream of TLR9 limits liver damage by upregulating IL-1 receptor antagonist [185]. Conversely, NOD2 ligation by gut-derived muramyl dipeptide drives IRF4 upregulation in liver pDCs, increases B7-H1 (PD-L1) expression, and selectively suppresses their IFN-α and IL-12/IL-6/TNF production and T-cell allostimulatory capacity, thereby imposing a tolerogenic brake that is specific to hepatic pDCs and dependent on IRF4–B7-H1 signaling [186]. IRF6 contributes an additional tolerance module; IRF6-deficient DCs show exaggerated TLR4-elicited NF-κB activation and IL-6/TNF secretion, identifying IRF6 as a negative regulator of DC inflammation [187]. IRF9 is expressed in hepatic DCs as part of the ISGF3 complex (STAT1–STAT2–IRF9) and integrates IFN-α/β signaling into IRG/ISG expression programs, particularly in cDC1, although direct IRF9-dominant functions in liver DCs remain insufficiently defined [188]. IRF2 also indirectly supports DC development by restraining excessive type I IFN exposure during early cDC differentiation, thereby preventing IFN-induced maturation defects [189]. Taken together, these data position DC-intrinsic IRF4/IRF8 as key determinants of cDC1/cDC2 balance, and IRF3/IRF7/IRF9 as the core type I IFN module. In the context of ALD, where gut-derived TLR7/9 ligands, CpG DNA and mito-DAMPs converge on hepatic DCs, this IRF network is likely to influence whether DC responses skew toward pathogenic Th17/neutrophilic immunity or toward IFN- and PD-L1-dependent tolerance, suggesting that selectively modulating IRF signaling in hepatic DCs may help to rebalance pathogenic and tolerogenic responses in ALD.

### 4.4. T Cells

T cells express a broad IRF repertoire centered on IRF1, IRF2, IRF3, IRF4, IRF5, IRF7, IRF8, and IRF9, each contributing to antiviral defense, inflammatory programming, or regulatory T-cell differentiation, whereas IRF6 has no convincing evidence of functional expression in T-cell subsets across human or murine datasets. IRF1 is the most extensively characterized and governs T-cell-mediated liver injury by promoting IFN-γ–dependent chemokine induction and cytotoxic programs [156,190], and its deficiency results in impaired antiviral T- and NK-cell responses [191]. IRF2 exerts an opposing, restraining influence by limiting type I IFN signaling during early T-cell development and enabling the generation of CD1d-independent, NK receptor–bearing T cells [192], while it also participates indirectly in hepatic immune regulation through PD-L1 modulation [193]. IRF3, though not a classical T-cell factor, contributes to hepatic immune pathology by amplifying type I IFN responses that shape T-cell recruitment, as seen in viral hepatitis and STING-driven liver inflammation [194]. IRF4 plays a lineage-defining role in Th2, Th9, Th 17 and Treg biology, controlling cytokine outputs and influencing susceptibility to parasitic and autoimmune liver injury and liver fibrosis [195,196,197]. IRF5 promotes Th1 differentiation [198], while IRF7 facilitates antiviral cytokine programs that are crucial for hepatic viral clearance [199]. Research evidence has shown that IRF8 may contribute to T-cell-dependent hepatic pathology through support of cytotoxic effector differentiation [168]. Moreover, IRF9 participates in IFN-stimulated gene induction in activated T cells and modulates hepatic inflammation in settings where IFN-JAK–STAT signaling is dominant [200]. Across liver diseases, these IRFs converge to regulate T-cell activation, trafficking, and effector functions in viral hepatitis, autoimmune hepatitis, ischemia–reperfusion injury, and tumor immunity. In ALD, elevated type I/II IFNs and cGAS–STING activation may reinforce IRF1/IRF4/IRF8/IRF9-dependent T-cell programs. However, direct evidence for IRF-regulated T-cell remodeling in ALD remains limited and largely extrapolated from other inflammatory liver models.

### 4.5. Hepatic Stellate Cells

Hepatic stellate cells (HSCs) express a restricted, but functionally important IRF repertoire dominated by IRF1, IRF2, IRF3, and IRF7, whereas IRF4, IRF5, IRF6, IRF8, and IRF9 lack direct evidence in HSCs. Among these factors, IRF1 is a potent driver of pro-inflammatory and pro-apoptotic programs in activated HSCs. Its upregulation enhances IFN-γRβ, Fas, and caspase signaling, thereby sensitizing HSCs to apoptosis and amplifying cytokine-driven liver injury [201,202]. IRF2 is present at high levels and contributes to lineage stabilization, exerting a supportive but non-dominant influence on HSC activation and phenotype regulation [203]. In contrast, IRF3 and IRF7 constitute the core antiviral machinery of HSCs, being strongly activated downstream of RIG-I, MDA5, and TLR3; HSCs stimulated via these pathways produce IFN-β and IFN-λ, which suppress HBV or HCV replication in neighboring hepatocytes [204,205,206]. IRF3 also contributes to HSC proliferative responses, as IRF3 silencing attenuates TGF-β1-induced HSC proliferation [207]. These antiviral and immunoregulatory roles position HSCs as unexpected contributors to hepatic innate immunity rather than passive ECM-producing cells. In the context of ALD, ethanol increases gut-derived dsRNA and mitochondrial DAMPs that activate TLR3, RIG-I, and cGAS–STING pathways, all of which converge on IRF3/IRF7 signaling. This suggests that ALD may directly activate the HSC interferon program [207]. IRF1-driven inflammatory signaling may further enhance HSC activation and fibrosis, whereas IRF3/7-dependent IFN-λ may influence hepatocyte injury and regeneration. Overall, current evidence supports a model in which HSC-intrinsic IRF1–IRF3–IRF7 circuits integrate antiviral, inflammatory, and fibrogenic signals. Although direct data in ALD remain limited, the convergence of TLR3/RIG-I/STING ligands in alcohol injury strongly implicates HSC-associated IRFs as active regulators of hepatic inflammation and fibrosis in alcohol-induced liver disease.

### 4.6. Neutrophils

Neutrophils are central effector cells in ALD, yet their regulation by IRF signaling is not well defined. Most mechanistic insights that link neutrophils to IRFs come from viral or sterile injury models rather than ALD-specific context. In ALD, hepatocytes and macrophages produce chemokines such as CXCL1, CXCL2, and IL-8, which drive substantial neutrophil recruitment to the liver [208,209]. Stressed hepatocytes further reinforce this process through LECT2-mediated chemotaxis [210]. In severe alcoholic hepatitis (sAH), IL-8^+^ neutrophils accumulate and form a self-reinforcing inflammatory circuit [209], paralleling NET-prone low-density neutrophils described in AH [211]. NETs (Neutrophil Extracellular Trap) directly damage hepatocytes and promote thrombosis [211], and neutrophil elastase can be inserted into hepatocytes to perturb Ca^2+^ signaling [212]. Although IRF-centered neutrophil programming is largely unstudied in ALD, upstream innate pathways that converge on IRFs clearly shape neutrophil behavior: TLR4, TLR7/9, and cGAS–STING activation enhance type I IFN and IRF3/7 signaling in hepatocytes and macrophages [185,213], thereby elevating CXCL10, type I IFN-stimulated genes, and downstream neutrophil recruitment. Notably, neutrophil-relevant IRF regulation has been demonstrated in non-hepatic inflammatory settings: IRF8 can induce intrinsic functional changes in mature neutrophils, selectively shaping LPS-driven inflammatory outputs [214], and neutrophils can couple nucleic-acid sensing to IRF-associated responses, including NET-linked interferon programs [215] and STING pathway-dependent inflammatory cell death in neutrophils [216]. NET formation, a key contributor to AH pathology [211,217], is known from sterile-injury models to be facilitated by IRF3–TBK1 activation, although this has not yet been directly validated in ALD. Platelet–neutrophil aggregates [218], which may enhance NETosis in ALD, also intersect with IRF-regulated inflammatory cytokines in macrophages, forming multi-cellular feedback circuits. Overall, current evidence indicates that neutrophil-driven hepatotoxicity is mediated through chemokine-mediated recruitment, NET-associated cytotoxicity, elastase-mediated hepatocyte damage, and altered granulopoiesis. However, the specific IRF-driven transcriptional programs within neutrophils during ALD remain largely undefined and warrant further investigation.

## 5. IRF-Targeted Modulators in Alcohol-Associated Liver Disease

Increasing evidence indicates that the immunopathology of ALD is tightly linked to dysregulated IRF signaling—most prominently the IRF3/IRF7 antiviral axis, the IRF1/IRF5 inflammatory module, and the IRF4/IRF8 balance controlling dendritic-cell and T-cell homeostasis. Several pharmacologic interventions currently investigated in ALD or ALD-relevant models modulate these IRF pathways either directly (e.g., TLR/STING/BTK blockade) or indirectly (e.g., IL-22 therapy, NETosis inhibition). In the following sections, we highlight therapeutic modalities with experimentally validated relevance to IRF-driven inflammation in ALD.

### 5.1. STING–TBK1–IRF3 Axis Inhibition

Mitochondrial injury caused by ethanol leads to leakage of mtDNA, activating cGAS–STING and driving TBK1-dependent phosphorylation of IRF3, an essential early event in alcoholic liver injury [17]. Persistent IRF3 activation contributes to hepatocyte apoptosis, mitochondrial collapse, and propagation of paracrine inflammatory signaling. Although STING inhibitors (e.g., H-151, C-176, C-178) have not yet been tested directly in ALD, their mechanistic relevance is clear: blocking STING-mediated IRF3 activation represents a rational strategy to attenuate early necroinflammatory events. Similarly, pharmacologic TBK1 inhibition (e.g., amlexanox) could theoretically dampen IRF3/IRF7 activation in hepatocytes and Kupffer cells. Given that elevated p-IRF3 correlates with AH severity, targeting this upstream kinase complex may provide a highly specific anti-inflammatory approach.

### 5.2. TLR4–IRF5 Inhibition via Humanized Anti-TLR4 Antibody

Among IRF family members, IRF5 is a dominant driver of M1 polarization, IL-1β/TNF-α secretion, and inflammatory tissue injury downstream of TLR7/8/9–MyD88 and TLR4 signaling. A humanized anti-TLR4 Fab fragment was shown to suppress LPS-induced IL-6, TNF-α, and chemokine production in vitro and in vivo [219]. Although not tested in ALD models, this antibody directly suppresses the upstream node driving IRF5 activation, suggesting potential clinical utility in limiting excessive macrophage activation and neutrophil recruitment in AH. Given that TLR4 is one of the central sensors in ALD pathogenesis [27], targeted inhibition of TLR4-IRF5 signaling could represent a mechanistically grounded strategy to limit pan-cytokine production and NET-associated immunothrombosis.

### 5.3. Pharmacologic Suppression of IRF4-Driven DC2/Th17 Responses

Experimental and clinical data indicate that IRF4 is a core transcriptional driver of cDC2 and Th17 programs, acting together with BATF/AP-1 at composite regulatory elements to induce IL-23 and IL-17-skewing signatures in mucosal and systemic inflammation [220,221]. Although IRF4 has not yet been directly targeted in ALD models, several pharmacologic approaches can attenuate IRF4-dependent circuits in DCs and T cells. JAK–STAT inhibitors, which dampen IL-6/IL-21/IL-23–STAT3 signaling, reduce Th17 differentiation and downstream IRF4-dependent effector programs in autoimmune settings [222,223], suggesting a plausible strategy to blunt intestinal and hepatic cDC2–Th17 expansion in ALD. Likewise, blockade of STAT3 with small-molecule inhibitors such as JQ1 diminishes IRF4-associated transcriptional modules in lymphocytes and myeloid cells [224], while BET bromodomain inhibitors such as I-BET762 downregulate IRF4 expression and IRF4-driven inflammatory genes in hematologic malignancies [225]. These data support a testable model in which pharmacologic interference with JAK–STAT3 axis could restrain IRF4-high cDC2 and Th17 responses in ALD, thereby mitigating IL-23/IL-17–mediated liver injury, even though direct validation of IRF4-targeted agents in ALD remains to be established.

### 5.4. IL-22 as an IRF-Modulating Cytoprotective Cytokine

IL-22 has emerged as a hepatoprotective cytokine in AH clinical investigations [226]. Mechanistically, IL-22 promotes STAT3 activation, enhances epithelial regeneration, protects against mitochondrial stress, and indirectly modulates IRF9-ISGF3 signaling by reducing IRF9-mediated pro-apoptotic transcription. In ethanol-stressed hepatocytes, IL-22 decreases oxidative stress and improves barrier integrity—two processes tightly intertwined with IRF-driven DAMP-triggered inflammation. Given that IRF9 amplification contributes to epithelial apoptosis in metabolic and oxidative injury [150], IL-22 may exert dual benefits: tissue protection and rebalancing of the IRF9–JAK–STAT axis.

### 5.5. BTK Inhibition to Restrict Granulopoiesis and NETosis (IRF5/IRF3 Axis)

Neutrophils are central effectors of AH pathology, and in vivo BTK inhibition markedly attenuates ALD through suppression of CD84-mediated granulopoiesis and improved neutrophil phenotype [227]. NET-prone low-density neutrophils, which accumulate in AH [211], further exacerbate hepatocyte injury and strongly correlate with disease severity and mortality. Because NETosis has been associated with TBK1–IRF3 activation in sterile injury models, BTK inhibition indirectly modulates IRF-driven inflammatory programming while simultaneously reducing neutrophil-mediated cytotoxicity. This represents one of the few interventions with direct in vivo evidence of ALD among IRF-relevant treatments.

### 5.6. NETosis Inhibitors: PAD4 Blockade and DNase I

NETs induce hepatocyte death, microvascular thrombosis, and amplify IL-1β/IL-8 loops in AH [211,227]. Although not traditionally viewed as IRF-targeting drugs, PAD4 inhibitors (e.g., Cl-amidine) and DNase I reduce NET formation downstream of IRF3/TBK1-mediated signals and attenuate multi-cellular inflammatory amplification. Given the strong causal link between NETs and ALD severity, incorporating NET-targeted therapy offers a downstream method to modulate IRF-primed neutrophil activation.

### 5.7. Senolytics Reducing Chronic IRF1/IRF3 Inflammation

Senescent hepatocytes accumulate during chronic alcohol exposure and sustain persistent IRF1/IRF3 activation. Senolytic therapy with dasatinib + quercetin (D+Q) improved liver injury, restored immune balance, and reduced chronic inflammatory signaling in ALD [228]. Because IRF1 and IRF3 are key transcriptional enforcers of senescence-associated inflammatory phenotypes, targeted senolysis offers a unique avenue to interrupt long-term IRF-driven damage.

### 5.8. Clinical Potential and Priority Research Directions

From a translational perspective, the therapeutic strategies discussed above differ substantially in their stage of development and potential clinical applicability. Among these approaches, IL-22–based therapy currently shows the strongest near-term translational signal, as it has entered clinical evaluation in alcoholic hepatitis and directly targets epithelial protection and tissue repair. Targeting macrophage-associated inflammatory amplification, such as BTK inhibition or modulation of TLR4–IRF5 signaling, may also be attractive for repurposing given existing human pharmacology and their relevance to cytokine-driven liver injury. In contrast, direct inhibition of upstream innate-sensing pathways, including the cGAS–STING–TBK1–IRF3 axis, is mechanistically compelling but requires careful consideration of host-defense impairment and infection risk, particularly in advanced ALD and AH. NET-directed approaches such as PAD4 inhibition or DNase I are conceptually suited as adjunctive strategies to limit NET-driven immunothrombosis and acute tissue injury, whereas senolytics represent an earlier-stage option that may be more relevant to chronic, low-grade IRF1/IRF3-associated inflammation. Priority research directions include defining stage-specific IRF activity across the ALD spectrum, developing biomarker-guided patient stratification (e.g., interferon/ISG signatures and circulating NET markers), and testing rational combination strategies that balance inflammatory control with preservation of antimicrobial immunity.

Taken together, these pharmacologic strategies underscore that IRF signaling functionally integrates mitochondrial damage, innate immune activation, DC2–Th17 skewing, neutrophil dysfunction, and chronic senescence in ALD. Upstream inhibition of the cGAS–STING–TBK1–IRF3 axis may attenuate early necroinflammatory injury, whereas targeting TLR4–IRF5 signaling provides a mechanistic route to restrain macrophage-driven cytokine amplification and NET-associated immunothrombosis. Modulation of IRF4-dependent cDC2/Th17 responses through JAK–STAT3 interference further illustrates how distinct IRF modules shape mucosal and hepatic IL-23/IL-17 circuits. Concurrently, IL-22 therapy, BTK inhibition, NET-directed approaches such as PAD4 inhibition or DNase I, and senolytic regimens address downstream IRF-linked epithelial stress, neutrophil cytotoxicity, and sustained inflammatory states. In this context, NET-directed approaches are conceptually suited as adjunctive strategies to limit NET-driven immunothrombosis and acute tissue injury, whereas senolytics represent an earlier-stage option that may be more relevant to chronic, low-grade IRF1/IRF3-associated inflammation. Although most agents remain untested directly in ALD, this framework highlights how IRF-centered pathways modulate multiple pathogenic layers, and how selectively attenuating these nodes may inform the rational prioritization of future therapeutic strategies, pending systematic validation in preclinical ALD models.

## 6. Conclusions and Prospects

ALD arises from a multilayered interaction among hepatocellular metabolic stress, innate immune activation, and gut–liver axis dysregulation. IRFs function as central transcriptional nodes integrating mitochondrial DAMPs, TLR signaling, cytokine cues, and metabolic perturbations into distinct inflammatory or reparative outputs. Across hepatic cell types, IRFs generally promote ALD progression by amplifying IFN-driven injury, chemokine production, PANoptosis, and neutrophil recruitment, whereas IRF2/IRF6 exhibit context-dependent protective roles, supporting immune resolution, metabolic balance, and epithelial integrity. Additionally, ALD also disrupts key lineage-defining IRF circuits—such as the IRF4/IRF8 axis in dendritic cells and the IRF1/IRF9 axis in hepatocytes—driving maladaptive Th17 immunity and DAMP amplification.

Recent studies have begun to define how IRF signaling operates in individual hepatic cell populations, including macrophages, dendritic-cell subsets, T cells, neutrophils, and stellate cells. These cell-type-specific pathways may determine whether the immune response evolves toward cytotoxic inflammation or toward controlled resolution. Further work is needed to map these circuits in human ALD and to understand how IRF-driven transcriptional states vary across disease stages and patient subgroups.

Therapeutically, several upstream pathways that converge on IRFs—such as cGAS–STING, TBK1, and TLR4—are now being explored as intervention points. Whether modulating IRF activity directly or indirectly can mitigate hepatocellular stress, correct immune dysregulation, or limit fibrogenesis remains an important question for future studies. As mechanistic insights continue to accumulate, defining how IRF-related signatures correspond to clinical phenotypes may help refine disease classification and guide the development of more selective therapeutic strategies for ALD.

## Figures and Tables

**Table 1:** DAMPs and PAMPs that activate PRR–IRF modules in ALD.

Origin	Representative DAMP/PAMP	Principal Receptor or Sensor	Likely IRF Modules Involved
**DAMPs**	Mitochondrial DNA	TLR9, NLRP3, cGAS-STING [81]	IRF3/IRF7 [29,51]
	Mitochondrial dsRNA	TLR3, MDA5 and RIG-I, PKR [82]	IRF3/IRF7 [63,82]
	HMGB1	TLR4, RAGE [83]	IRF3 [83]
	Nuclear DNA	TLR9 [84]	IRF3/IRF7 [85]
	MicroRNA	RISC [86], TLRs [87]	IRFs [88]
	Extracellular vesicles	NLRP3 [89], LDLR [90], ASGPR [91], NR2 [92], TLRs [93,94]	IRF3/IRF7 [65,95]
	Lipids (FFA, TG)	CD36 [96], GPR120/40 [97,98], PPAR [99], TLRs [100]	IRF3/IRF7/IRF6 [100,101]
	4-HNE/MDA	TRPA1 [102], GPR109A [103] TLR2/4 [104,105]	not yet clear
	Extracellular ATP/adenosine	P receptors [106]	not yet clear
**PAMPs**	LPS	CD14 [107], TLR2/4 [108,109]	IRF3/IRF7 [108]
	Bacterial DNA	TLR9 [73]	IRF3/IRF7
	Microbe-derivede xtracellular vesicles	TLR2/4/8 [110,111]	IRF3/IRF7 [112,113]
	β-glucan	CLEC7A/Dectin-1 [76,114]	IRF3/IRF5 [115,116]
	Lipoteichoic acid	CD14, TLR2 [117]	IRF2 [118]
	flagellin	TLR5 [75]	IRF3/IRF7 [119]

## Data Availability

No new data were created or analyzed in this study. Data sharing is not applicable to this article.

## Abbreviations

ADH	alcohol dehydrogenase
AH	alcohol-associated hepatitis
ALD	alcohol-associated liver disease
cGAS	cyclic GMP–AMP synthase
CYP2E1	cytochrome P450 2E1
DAMP	damage-associated molecular pattern
DC	dendritic cell
dsRNA	double-stranded RNA
RIG-I	retinoic acid-inducible gene I
HSCs	hepatic stellate cells
IFNs	interferons
IKKε	inhibitor of nuclear factor kappa-B kinase ε
ISG	interferon-stimulated gene
KCs	Kupffer cells
MAVS	mitochondrial antiviral signaling protein
MDA5	melanoma differentiation-associated protein 5
MyD88	myeloid differentiation primary response 88
mtDNA	mitochondrial DNA
PAMP	pathogen-associated molecular pattern
PRR	pattern-recognition receptor
PTM	post-translational modification
ROS	reactive oxygen species
ssDNA	single-stranded DNA
ssRNA	single-stranded RNA
STING	stimulator of interferon genes
TBK1	TANK-binding kinase 1
TRIF	TIR-domain-containing adapter-inducing interferon-β
